# Raman Amplification Optimization in Short-Reach High Data Rate Coherent Transmission Systems †

**DOI:** 10.3390/s21196521

**Published:** 2021-09-29

**Authors:** Mingming Tan, Md Asif Iqbal, Tu T. Nguyen, Paweł Rosa, Lukasz Krzczanowicz, Ian. D. Phillips, Paul Harper, Wladek Forysiak

**Affiliations:** 1 Aston Institute of Photonic Technologies, Aston University, Birmingham B4 7ET, UK; nguyentu.hcmuns@gmail.com (T.T.N.); lukkr@fotonik.dtu.dk (L.K.); i.phillips@aston.ac.uk (I.D.P.); p.harper@aston.ac.uk (P.H.); W.Forysiak@aston.ac.uk (W.F.); 2 BT Applied Research, Adastral Park, Ipswich IP5 3RE, UK; mdasif.iqbal@bt.com; 3 Infinera Pennsylvania, 7360 Windsor Dr, Allentown, PA 18106, USA; 4 National Institute of Telecommunications, 04-894 Warsaw, Poland; P.Rosa@il-pib.pl; 5 Department of Photonics Engineering, Technical University of Denmark, 2800 Kgs. Lyngby, Denmark

**Keywords:** Raman amplification, optical fibre communication

## Abstract

We compared the transmission performances of 600 Gbit/s PM-64QAM WDM signals over 75.6 km of single-mode fibre (SMF) using EDFA, discrete Raman, hybrid Raman/EDFA, and first-order or second-order (dual-order) distributed Raman amplifiers. Our numerical simulations and experimental results showed that the simple first-order distributed Raman scheme with backward pumping delivered the best transmission performance among all the schemes, notably better than the expected second-order Raman scheme, which gave a flatter signal power variation along the fibre. Using the first-order backward Raman pumping scheme demonstrated a better balance between the ASE noise and fibre nonlinearity and gave an optimal transmission performance over a relatively short distance of 75 km SMF.

## 1. Introduction

In unrepeatered coherent transmission systems, distributed Raman amplification (DRA) can provide a better signal-to-noise ratio (SNR) than lumped (EDFA, discrete Raman amplification) or hybrid amplification techniques (hybrid Raman/EDFA) [1,2,3,4,5,6,7]. Particularly, higher-order DRA (second-order) has been advantageous in the transmission performance compared with first-order DRA because it minimises the signal power variation along the fibre and demonstrates a better balance between the amplified spontaneous emission (ASE) noise and the Kerr nonlinearities of the optical fibre in long-haul transmission systems [4]. However, as the data capacity of the optical transceiver has been increased from 100 Gb/s PM-QPSK to 600 Gb/s PM-64QAM, the maximum reach has significantly decreased from several thousand kilometres with QPSK to metro-network or data-centre-interconnect (DCI) with 64QAM [8,9], which can usually go up to a hundred kilometres or more. In such applications, the optimisation of the amplification technique remains to be investigated, so we would like to determine which amplification scheme delivers superior transmission performance over a relatively short fibre length with dual-polarisation 69 GBaud 64QAM signals.

In this paper, we expand our work on the optimisation of amplifiers in [10] and numerically and experimentally evaluate the performances of different representative amplification techniques used for transmission over 75.6 km SMF. The following discrete, hybrid, and distributed optical amplifiers are considered: EDFA, discrete Raman amplification, hybrid Raman/EDFA, first-order Raman-only amplification, and second-order (dual-order) distributed Raman amplification. First, we characterise the signal and noise power profiles of each scheme numerically and experimentally, and then we conduct the measurement of optical signal-to-noise ratio (OSNR) with 1 channel and 11 channels. It is shown that the noise level of the second-order DRA is indeed slightly lower than that in the other schemes. Finally, we experimentally evaluate how optical amplifiers perform using an 11-channel WDM grid with a 600 Gbit/s (69.4 Gbaud, 832 Gbit/s line rate) PM-64QAM real-time transceiver over 75 km SMF. Although our OSNR characterisations show that the second-order distributed Raman amplification had the lowest ASE noise level, the first-order distributed Raman amplification gave the best transmission performance, demonstrating the optimum balance between the linear noise and the fibre nonlinearities. Based on the simulated signal and noise power profiles, our transmission simulations show the same results as the experiment. For a short-reach metro network or DCI application with high-data-rate transceivers, the distributed Raman amplifier delivered the best transmission performance, compared with any other amplification scheme, including hybrid Raman/EDFA, discrete Raman, and EDFA only. However, the first-order distributed Raman scheme, with a simpler setup and lower pump power, performed better than the second-order scheme.

## 2. Experimental Setup and Characterization of All Amplification Schemes

**Figure 1:**
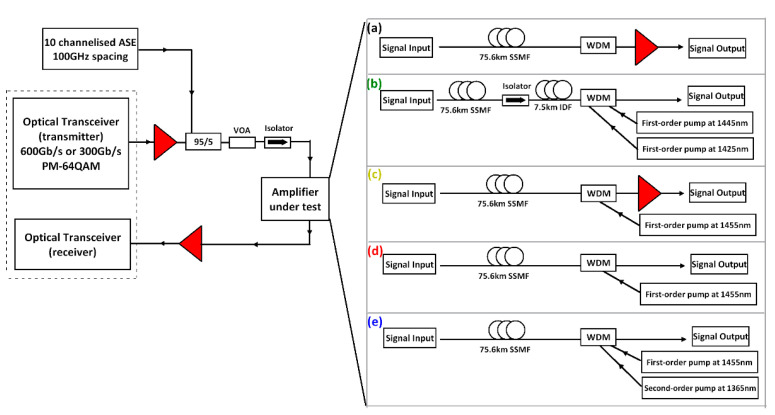
Experimental setup showing high-data-rate transmission with different amplification schemes: (**a**) EDFA only; (**b**) discrete Raman amplifier; (**c**) hybrid Raman/EDFA; (**d**) first-order distributed Raman amplifier; (**e**) second-order distributed Raman amplifier.

Figure 1 shows our experimental setup. We conducted an unrepeated single-span experiment to evaluate the transmission performances of different optical amplification schemes using a high-data-rate signal. A commercially available transceiver was used to generate a 69.4 Gbaud polarization-division-multiplexed (PDM)-64QAM signal (50 Gbaud for the signal and the remainder for the FEC and other overheads), or a 34.7 Gbaud PDM-64QAM signal, corresponding to 600 Gbit/s and 300 Gbit/s data capacity, respectively. Ten channelized ASE signals spaced at 100 GHz (ranging from 1543.73 nm to 1551.72 nm) and the PDM-64QAM modulated signal centred at 1547.72 nm were combined via a 95/5 coupler to give an 11-channel WDM grid. The output signal was amplified by an EDFA and attenuated by a variable optical attenuator (VOA) to adjust the signal launch power to the fibre span. The amplified fibre link was a 75.6 km SMF with a WDM coupler (~0.8 dB attenuation) which gave a total attenuation of ~15.3 dB. The output signal from the link was amplified by an EDFA and then attenuated by a VOA in order to maintain a constant input power of −5 dBm to the receiver. The built-in real-time DSP algorithm was used to compensate for the linear impairments, and the BERs were calculated over 1 trillion (10^12) bits.

**Figure 2:**
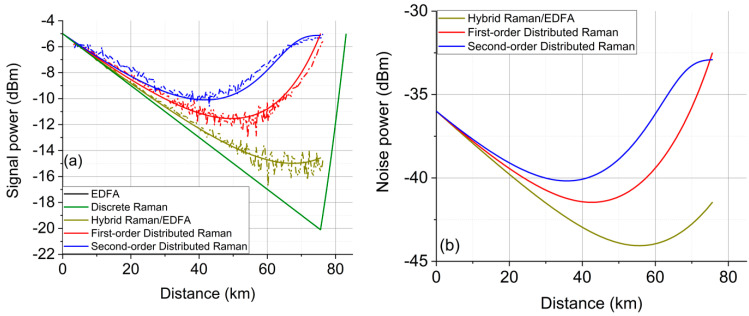
(**a**) Experimentally measured (dotted line) and simulated (solid line) signal power profiles along the fibre with five different amplification schemes; (**b**) Simulated noise power profiles along the fibre.

There were five amplification schemes investigated over the single 75.6 km SMF span, as illustrated on the right side of Figure 1, and the experimentally measured and simulated signal power profiles along the fibre are shown in Figure 2a. Scheme (a) was EDFA only to compensate the overall ~15 dB loss from the fibre and the WDM coupler, where the signal power in dBm decreased linearly along the fibre as shown in Figure 2a and amplified within the last few metres of the fibre. Scheme (b) was a discrete Raman amplifier that used two first-order Raman pumps at 1425 nm (~320 mW pump power) and 1445 nm (~350 mW pump power). Similarly, this is also a lumped amplification scheme, but 7.5 km of inverse dispersion fibre (IDF) was used as the Raman gain medium [5]. The main drawback of discrete Raman amplification in scheme (b) was the relatively long length of the gain fibre (7.5 km in this case), causing higher loss and higher accumulated fibre nonlinearity, compared to only a few metres in the EDFA case. Figure 2b clearly illustrates that the signal power using the discrete Raman amplifier decreased linearly, as was observed with the EDFA, but the amplification started within the last ~8 km. This extra length of Raman gain fibre could potentially increase both the linear ASE noise and the fibre nonlinearity when conducting the signal transmission. Scheme (c) was a hybrid amplification scheme combining the DRA and EDFA, which used first-order backward (BW) Raman pumping and an EDFA [6,9]. The first-order DRA provided ~5 dB Raman gain with 160 mW pump power at 1455 nm, and the EDFA provided ~10 dB gain. Thus, as demonstrated in Figure 2a, the signal power variation along the fibre was ~10 dB compared with 15 dB using the EDFA-only or discrete Raman scheme. Scheme (d) was a distributed Raman amplifier using a first-order BW-propagated Raman pump at 1455 nm. The pump power at 1455 nm was ~410 mW, giving a signal power variation of ~6.5 dB. Scheme (e) was effectively a dual-order BW Raman pumping scheme, which used a second-order 1365 nm pump in addition to the 1455 nm seed pump. The pump power was ~940 mW at 1365 nm and ~25 mW at 1455 nm, giving a total pump power that was significantly higher than that in the other schemes but giving only ~4 dB signal power variation, which was the lowest of all the amplification schemes tested [2,3,10,11].

Figure 2b shows the simulated noise power profiles along the fibre for schemes (c), (d), and (e) from Figure 1. In EDFA and discrete Raman schemes (Figure 1a,b), the fixed ASE noise was added at the end of the fibre. In the discrete Raman amplifier, the noise was generated and accumulated over the last 8 km of the gain fibre, resulting in the highest noise power of all schemes—even higher than the EDFA scheme [12]. The hybrid Raman/EDFA was partially a distributed Raman amplifier where the noise was distributed over the whole transmission fibre, with the exception of EDFA noise which was added at the very last point of the fibre, increasing the overall noise level. From the two distributed schemes, the noise level of the second-order scheme was higher than that of the first-order scheme for the first ~73 km; however, within the last 2 km, the noise power of the first-order scheme dramatically exceeded the noise of the second-order scheme.

**Figure 3:**
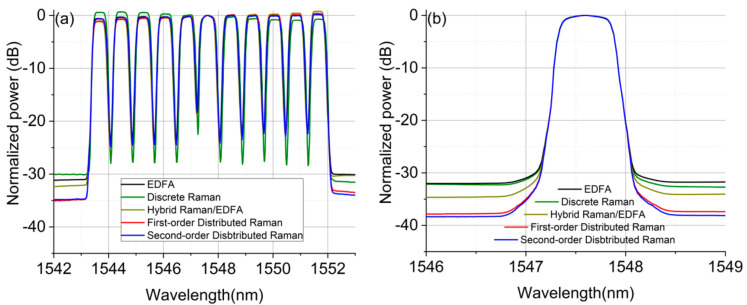
The output spectra from different optical amplifiers over a 75.6 km SMF: (**a**) with 11-channel input; (**b**) with 1 channel input.

Figure 3 compares WDM (a) and a single channel (b) output signal spectra for all amplification schemes after the 75.6 km transmission span. The two DRA schemes showed significantly lower ASE noise levels (potentially higher signal-to-noise ratio) compared with the other three schemes, by approximately 5–6 dB, which means that the transmission performance in the linear regime is likely to show the same trend. The second-order distributed Raman scheme (blue) performed the best in terms of received OSNR, followed by the first-order scheme (red) with a slightly higher noise level as numerically shown in Figure 2b. This means that the superior noise performance using the second-order pumping was not obvious in the single 75.6 km span.

## 3. Results and Discussion

**Figure 4:**
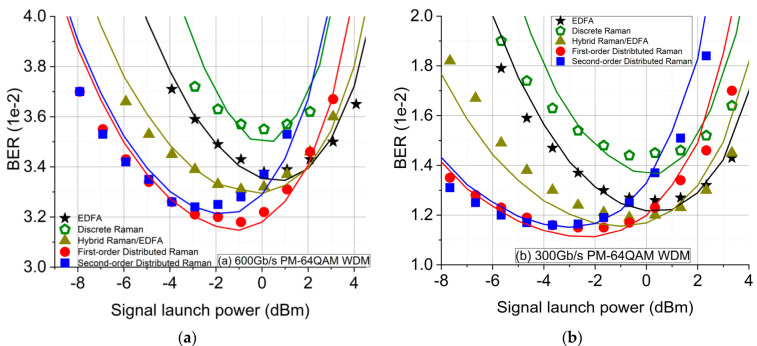
Simulated and experimental BER performances over a 75.6 km SMF with different amplification schemes measured at the centred channel (at 1547.72 nm): (**a**) 600 Gb/s PM-64QAM WDM transceiver; (**b**) 300 Gb/s PM-64QAM WDM transceiver.

The transmission performance was tested experimentally using a PM-64QAM WDM signal centred at 1547.72 nm and confirmed through simulation with signals of 600 Gb/s (Figure 4a) and 300 Gb/s (Figure 4b). We conducted a numerical simulation of the transmission performance of the PM-64QAM system, taking into account the simulated signal and noise power profiles for each amplification scheme. The simulation setup was similar, a PRBS length of 2^16^-1 was used instead of 2^15^-1. The propagation of the dual-polarisation complex envelope of an optical signal in optical fibre is governed by the coupled nonlinear Schrödinger equations (Manakov equations) and was simulated using the well-known split-step Fourier method [13,14], with a step size of 0.3 km using the simulated signal power profiles shown in Figure 2a. The noise from EDFAs implemented in the experiments at the transmitter and the receiver was taken into account in the simulations (ASE noise power density for each EDFA was approximately −155 dBm/Hz). To emulate EDFA noise, Gaussian noise with PSD of −144 dBm/Hz and −149 dBm/Hz was added at the end of the fibre for EDFA-only and hybrid Raman/EDFA, respectively. In addition, for the two distributed schemes, Raman noise was simulated as Gaussian noise, which was added to the signal after each step (0.3 km), aligning with the simulated noise profiles shown in Figure 2b. We used the same signal and noise power profiles for all the signal launch powers for simplicity. At the receiver, after coherent detection, the channel under test was filtered using an ideal low-pass filter. In order to take the imperfection of the DSP chain used in the experiments into account, the simulation results were also normalized by the same amount of the experimentally maximum achievable SNR, which was fixed at 20 dB.

Figure 4a,b shows the BERs versus signal launch power for 600 Gbit/s and 300 Gbit/s 64QAM signals. The solid lines are the numerical simulation results, and the dots are experimental results. The BERs at the optimum powers for EDFA, discrete Raman, and hybrid Raman/EDFA agree with the noise level analysis above; of these three schemes which used lumped amplification, the hybrid scheme showed the best BER as being more distributed, followed by the EDFA, and the discrete Raman scheme gave the worst BER performance due to the long Raman gain fibre at the end of the span. All three of these schemes performed significantly worse than the two distributed Raman schemes. The first-order Raman scheme showed better transmission performances at the optimum signal launch power for both signals and data rates. In the linear regime, the BERs with the second-order Raman scheme almost overlapped with the first-order Raman scheme. This means that the benefit from using the higher-order Raman pumping was not revealed in such a short transmission distance because the transmission performance in the linear regime was also influenced by the noise from the transceiver [7] and the EDFAs at the transmitter and the receiver. However, in the nonlinear regime, the first-order scheme gave a larger signal power variation along the fibre, as shown in Figure 3a, and consequently had a lower average signal power. Therefore, the first-order DRA scheme showed significantly greater tolerance against the fibre nonlinearity and better transmission performances than the second-order DRA. The experimental results were confirmed with our numerical simulations, as illustrated in Figure 4. As for short-reach high-data-rate coherent transmission systems, the simple first-order distributed Raman scheme, requiring low pump power, gave the best transmission performance compared with any other scheme, including the dual-order Raman scheme, hybrid Raman/EDFA, discrete Raman, and EDFA-only schemes.

## 4. Conclusions

In this paper, we demonstrate the experimental and numerical characterisation and optimisation for representative optical amplifiers, including an EDFA, a discrete Raman amplifier, a hybrid Raman/EDFA, first-order only, and second-order (dual-order) distributed Raman amplifiers, with a 600 Gb/s PM-64QAM transceiver (11-channel WDM grid) over a 75.6 km SMF. Our stand-alone characterisation results demonstrate that the second-order Raman scheme had flatter signal power profiles along the fibre, the lowest ASE noise level, and the highest OSNR. However, in the experimental transmission test, the first-order distributed Raman amplifier gave the best overall transmission overall performance. In the linear regime, the improvement introduced by higher-order pumping was not apparent, and therefore the first-order scheme showed similar performance to the second-order Raman scheme. However, because of the lower average signal power, the first-order scheme showed significantly superior transmission performance in the nonlinear regime in comparison with the second-order scheme. Therefore, the simpler first-order scheme gave the optimum balance between the linear noise and fibre nonlinearities in a single-span system with a high-data-rate transceiver. In addition, both distributed schemes demonstrated better BERs than the hybrid and discrete schemes. As expected, the hybrid Raman/EDFA scheme showed better performance than discrete schemes. Due to the extra 7.5 km Raman gain fibre, the discrete Raman scheme performed worst among all the amplification schemes considered.

## Data Availability

Not applicable.
